# Revisiting Hazard Ratios: Can We Define Causal Estimands for Time‐Dependent Treatment Effects?

**DOI:** 10.1002/bimj.70100

**Published:** 2025-11-29

**Authors:** Dominic Edelmann

**Affiliations:** ^1^ Division of Biostatistics German Cancer Research Center Heidelberg Germany

## Abstract

In this paper, some aspects concerning the causal interpretation of hazard contrasts are revisited. It is first investigated, in which sense the hazard ratio constitutes a causal effect. It is demonstrated that the hazard ratio at a timepoint t represents a causal effect for the population at baseline, but in general not for any population at risk at time t. Moreover, the scenario is studied, in which the survival curves coincide up to some timepoint t and then separate. This investigation provides valuable insight both on the causal interpretation of the conventional hazard ratio and on properties of the recently proposed causal hazard ratio. The findings suggest that, without making further assumptions, there is in general no meaningful estimand for a treatment effect at time t>0. It is therefore advocated to develop alternative estimands grounded in medically plausible assumptions about the joint distribution of counterfactual survival times.

## 1 Introduction

The Cox proportional hazards regression (Cox 1972) remains by far the most widely used model for time‐to‐event outcomes, and the hazard ratio has consequently become one of the most frequently reported effect measures in medical research. The very term “effect measure” implicitly suggests a causal interpretation, and it is common practice to treat changes in the hazard ratio over time as if they directly reflected time‐dependent treatment effects (Jachno et al. 2022; Fay and Li 2024). Such interpretations have long been recognized as problematic (Keiding 1999). More recent work (Hernán 2010; Martinussen et al. 2020; Martinussen 2022) has sharpened these concerns, showing that even in a randomized trial, a change in the hazard ratio does not necessarily reflect a change in the treatment effect. This work is motivated from the article by Beyersmann et al. (2025).

Fifteen years after Hernán's landmark paper, “The hazards of hazard ratios,” (Hernán 2010) the article by Beyersmann et al. (2025)—and the special issue surrounding it—may well mark a turning point for the inference of time‐dependent effects. While until now, the literature has mainly focused on describing the pitfalls of interpreting the hazard ratio as a causal treatment effect, Beyersmann et al. (2025) proposed a more pragmatic course, offering guidance for a cautious yet meaningful causal interpretation of hazard contrasts.

Most authors agree that on one hand the hazard ratio represents—at least in a certain sense—a causal effect and on the other hand can lead to counterintuitive conclusions when naively interpreted as treatment effect at a certain time point. Yet there is still substantial confusion and apparent dissonance in the literature. One reason for this confusion is that researchers often fail to distinguish properly between (1) whether the hazard ratio mathematically represents a causal effect and (2) whether it admits a meaningful or intuitive causal interpretation. This conflation of mathematical and semantic issues leads to misunderstandings. Indeed, some papers do not even define the term “causal effect,” making any strict logical analysis impossible. In this paper, such confusion is avoided by separating mathematical results from their interpretation as clearly as possible. The mathematical setup for our discussion is described in the following paragraph.

### 1.1 Mathematical Setup

Assumptions 1 Let (Ω,F,P) be a probability space and let $T_{0}:\Omega \to \left(0,\infty \right)$ and $T_{1}:\Omega \to \left(0,\infty \right)$ denote two measurable functions on (Ω,F) representing counterfactual survival outcomes. $T_{0}$ and $T_{1}$ are both assumed to be continuous with density functions $f_{0}\left(t\right)$ and $f_{1}\left(t\right)$, respectively. For i=1,2, the cumulative distribution functions is denoted by $F_{i}\left(t\right)$, the survival functions by $S_{i}\left(t\right)=1-F_{i}\left(t\right)$ and the hazard functions by

$$h_{i}\left(t\right)=\lim_{\Delta t↘0}\frac{P(t<T_{i}\leq t+\Delta t|T_{i}\geq t)}{\Delta t}=\frac{f_{i}\left(t\right)}{S_{i}\left(t\right)}.$$

Moreover, it is assumed that there is an interval [0,τ] such that

$$f_{0}\left(t\right)>0,f_{1}\left(t\right)>0\;\;\text{for all}\;t\in \left[0,\tau \right].$$

The (conventional) **hazard ratio** (HR) between $P_{T_{1}}$ and $P_{T_{0}}$ at time t∈[0,τ] then exists and is given by

(1) $$\text{HR}\left(t\right)=\frac{h_{1}\left(t\right)}{h_{0}\left(t\right)}.$$

Avoiding discussions about identifiability and estimation, we will take an “all‐knowing” population perspective in this paper, meaning that the joint distribution of $\left(T_{0},T_{1}\right)$ is fully known. For this reason, no assumptions on randomization of treatment are required and the introduction of a treatment variable is omitted. Yet it makes sense to read this paper with a situation of a randomized trial in mind allowing for identification of the hazard ratio in Equation (1).

### 1.2 Outline

In Section 2, we revisit the question in which sense the hazard ratio represents causal effect offering a simple and concise solution: for any t∈[0,τ], HR(t) is a causal effect for the full population at baseline (t=0), but not for a meaningful subpopulation at risk at time t. Consequently, interpretation of HR(t) as time‐dependent treatment effect is questionable.

In Section 3, we take a closer look at the case where the survival curves coincide up to some timepoint u. Although often dismissed as trivial, this setting provides valuable insight both on the causal interpretation of the conventional hazard ratio and on properties of the recently proposed causal hazard ratio (Martinussen et al. 2020; Martinussen 2022).

The findings of these sections suggest that—without additional structure—there is no generally useful or coherent definition of a treatment effect at time t>0 and that assumptions on the dependence structure of $T_{0}$ and $T_{1}$ are required to enable inference for time‐dependent treatment effects. The paper concludes with an outlook on future steps to establish statistical methods to infer time‐dependent treatment effects in Sections 4 an 5.

Throughout this comment, the hazard ratio is exemplarily treated, all results similarly hold for the hazard difference or other time‐dependent hazard contrasts; the described issues are not related to the non‐collapsibility of the hazard ratio (Aalen et al. 2015).

## 2 Causal Effect Properties of the Hazard Ratio

### 2.1 Motivation

There is substantial confusion in the literature about the question in which sense the hazard ratio HR(t) in Equation (1) represents a causal effect. In this section, a simple and concise answer to this question is provided: for any t>0, HR(t) is a causal effect for the full population at baseline (t=0), but it is not a causal effect for any subpopulation

$$A_{t}\subseteq \left\{T_{0}\geq t,T_{1}\geq t\right\}.$$

The subsets $A_{t}$ in turn are the only sensible subsets to consider when defining a treatment effect for a population at time t; there appears to be no reasonable way to define a treatment effect at time t for populations in which one (or both) counterfactual survival times can be smaller than t.

The subsequent arguments rely on the notion of a population causal effect introduced in Definition 2.2. This definition formalizes the characterization of such an effect as “any contrast between functionals of the marginal distributions of counterfactual outcomes” (Hernán and Robins 2010, p. 7). The reader is reminded that the general mathematical setup for this article has already been provided in Section 1.1.

### 2.2 Mathematical Formulation

Definition 2.1 For any probability measure Q on a measurable space (Ω,F), let $Q_{X}$ denote the induced probability measure, that is, $Q_{X}\left(A\right):=Q\left(X^{-1}\left(A\right)\right)$ for A∈F.

Definition 2.2 Let P denote a set of probability measures on a measurable space (Ω,F). Let $Y_{0}$ and $Y_{1}$ denote two measurable functions denoting counterfactual outcomes on (Ω,F) with $P_{Y_{0}},P_{Y_{1}}\in P$. Let ϕ:P→R denote some functional and f denote a contrast function $f:R^{2}\to R$. Then

$$\text{CE}\;\left(P_{Y_{1}},P_{Y_{0}}\right)=f\;\left(\phi \left(P_{Y_{1}}\right),\phi \left(P_{Y_{0}}\right)\right)$$

is called a **population causal effect** for comparing $Y_{1}$ and $Y_{0}$ under the probability distribution P.

The restriction to real‐valued functionals and contrast functions is made for reasons of simplicity. In Appendix A, a proposition for a more general definition of causal effects is attempted.

Remark 2.3 The attentive reader may have realized that the precise definition of the term *contrast function* has been omitted. To avoid unnecessary technical discussions, we avoid an exact definition of this term. For the following results, one only requires the following properties:

1. The difference f(x,y)=x−y and the ratio f(x,y)=x/y are contrast functions.
2. For each contrast function f, there is an element $c_{0}\in \left\{0,1\right\}$, such that $f\left(x,x\right)=c_{0}$ for all x∈R.

Proposition 2.4 Under Assumptions 1, for each t∈[0,τ], the hazard ratio HR(t) is a population causal effect for comparing $T_{0}$ and $T_{1}$ under P.

Let P be a suitably chosen set (e.g., $P=\{P_{T_{0}},P_{T_{1}}\}$). For probability measures Q with density function g and cumulative distribution function G, set

$$\phi \left(Q\right)=\frac{g(t)}{1-G(t)}\;\;f\left(x,y\right)=x/y.$$

 
□

Proposition 2.5 Under Assumption 1, for t∈(0,τ], HR(t) is in general **not** a population causal effect for comparing $T_{0}$ and $T_{1}$ under $P(\cdot |A_{t})$ for any subset

$$A_{t}\subseteq \left\{T_{0}\geq t,T_{1}\geq t\right\}$$

with $P(A_{t})>0$.

The statement of Proposition 2.5 can be shown by providing a simple counterexample. To avoid redundancy, Example 1 in Section 3 is used. Set t>u. For each

$$A_{t}\subseteq \left\{T_{0}\geq t,T_{1}\geq t\right\},$$

the distributions of $T_{1}$ and $T_{0}$ under $A_{t}$ are equal implying that any causal effect for comparing $T_{0}$ and $T_{1}$ under $P(\cdot |A_{t})$ is 0 or 1 (cf. Remark 2.3). On the other hand, 0<HR(t)<1.□

To simplify notation, we will often refer to a population causal effect under $P(\cdot |A_{t})$ as a *causal effect for the population* $A_{t}$, a notion that we have already informally used before.

Corollary 2.6 The (conventional) hazard ratio HR(t) is in general not equal to the **causal hazard ratio** ${\text{HR}}_{C}\left(t\right)$ defined as (Martinussen et al. 2020; Martinussen 2022)

$${\text{HR}}_{C}\left(t\right)=\lim_{\Delta t↘0}\frac{P(t<T_{1}\leq t+\Delta t|T_{0}\geq t,T_{1}\geq t)}{P(t<T_{0}\leq t+\Delta t|T_{0}\geq t,T_{1}\geq t)}.$$

### 2.3 Interpretation and Connection to Literature

We have established that HR(t) is a causal effect for the population at baseline and consequently represents a population treatment effect in the sense that it is a contrast between the distributions of the counterfactuals $T_{0}$ and $T_{1}$. A useful conclusion is that, if HR(t)≠1 for some t∈[0,τ], then the distributions of $T_{0}$ and $T_{1}$ are not equal.

On the other hand, if one would like to interpret a causal effect measure as a *population treatment effect at time* t, it appears natural to require that the population on which the corresponding causal effect is defined is at risk at time t and that both counterfactual survival times occur after t. HR(t) is in general not a causal effect of a population guaranteed to be at risk at time t and hence should not be naively interpreted as a treatment effect a time t.

Moreover providing an intuitive interpretation of the causal effect HR(t) in the general case appears very difficult, particularly when HR(t) is treated as a stand‐alone measure. A better explanation may be possible when contrasting the hazard functions in a functional sense, as proposed by Beyersmann et al. (2025).

While this section offers a strikingly simple perspective on the subtleties concerning the causality of the hazard ratio, the core findings of this section are of course not entirely new. Hernán and Robins (2010), e.g., clearly stated that the hazard ratio is a causal effect, but its “causal interpretation is not straightforward”, providing an example where HR(t)<1 indicates a beneficial treatment effect, while treatment is in fact harmful. Likewise, the conclusion by Martinussen (2022) that “the hazard ratio obtained cannot be given a causal interpretation when interpreted as a hazard ratio” is closely connected to our finding that HR(t) is not a causal effect at time t (cf. Corollary 2.6).

## 3 The Curious Case of Equal Survival Curves

### 3.1 Motivation

Many articles on the causal interpretation of the hazard ratio devote a brief remark to the setting, where the survival curves coincide up to a time u and then diverge. The prevailing view is that HR(t) does have causal interpretation in this case and that pitfalls such as highlighted in Section 2 are irrelevant. For instance, Hernán and Robins (2010, p. 225) argued that the “built‐in selection bias of hazard ratios does not happen if the survival curves are the same in the treated and the untreated”, and Beyersmann et al. (2025) concurred that interpreting the treatment is “ineffective on one time interval and beneficial on the other” is “certainly correct” in this setting.

In what follows we revisit this scenario in depth and show that it is far more intricate than these brief summary comments suggest. The literature typically restricts—explicitly or implicitly—to the trivial scenario

$$\left\{T_{0}\geq t\right\}=\left\{T_{1}\geq t\right\}\;\text{for all}\;t\leq u,$$

and neglects the general case

$$P\left(T_{0}\geq t\right)=P\left(T_{1}\geq t\right)\;\text{for all}\;t\leq u.$$

Using concrete counter‐examples, it is shown that when the latter equality holds, HR(t) is not always a causal effect for a subset of $\left\{T_{0}\geq t,T_{1}\geq t\right\}$. Moreover, it is demonstrated that the causal hazard ratio ${\mathrm{HR}}_{C}\left(t\right)$ (or any other causal effect for a subpopulation of $\left\{T_{0}\geq t,T_{1}\geq t\right\}$) is not necessarily a satisfying stand‐alone measure for the treatment affect at time t. In particular, there exist settings where ${\mathrm{HR}}_{C}\left(t\right)=1$ for all t>0, but the corresponding survival curves $S_{0}\left(t\right)$ and $S_{1}\left(t\right)$ differ for all values t>u. Even more there exists examples, where the causal hazard ratio implies a benefit of treatment, i.e., ${\mathrm{HR}}_{C}\left(t\right)<1$ for t≥u, but the survival curves suggest that treatment is harmful, i.e., $S_{0}\left(t\right)>S_{1}\left(t\right)$ for t≥u. Finally, we show that there are situations in which ${\mathrm{HR}}_{C}\left(t\right)$ is not even defined for all t in the joint support of $T_{0}$ and $T_{1}$.

These findings reveal that the “equal‐until‐u” case, far from being trivial, exposes fundamental limitations of both the conventional and causal hazard ratios. These shortcomings indicate that any attempt to infer time‐dependent treatment effects must be grounded in additional structural assumptions.

### 3.2 Construction of Counterexamples

For the first two examples, we assume that there are three subpopulations of equal size. This may be modeled by a random variable S, with P(S=1)=P(S=2)=P(S=3)=1/3. The survival functions in the three subdistributions will be denoted by $S_{i|S=s}\left(t\right)=P\left(T_{i}>t|S=s\right)$, i=0,1, s=1,2,3. Moreover for ease of notation, we define u=log(2).

**FIGURE 1:**
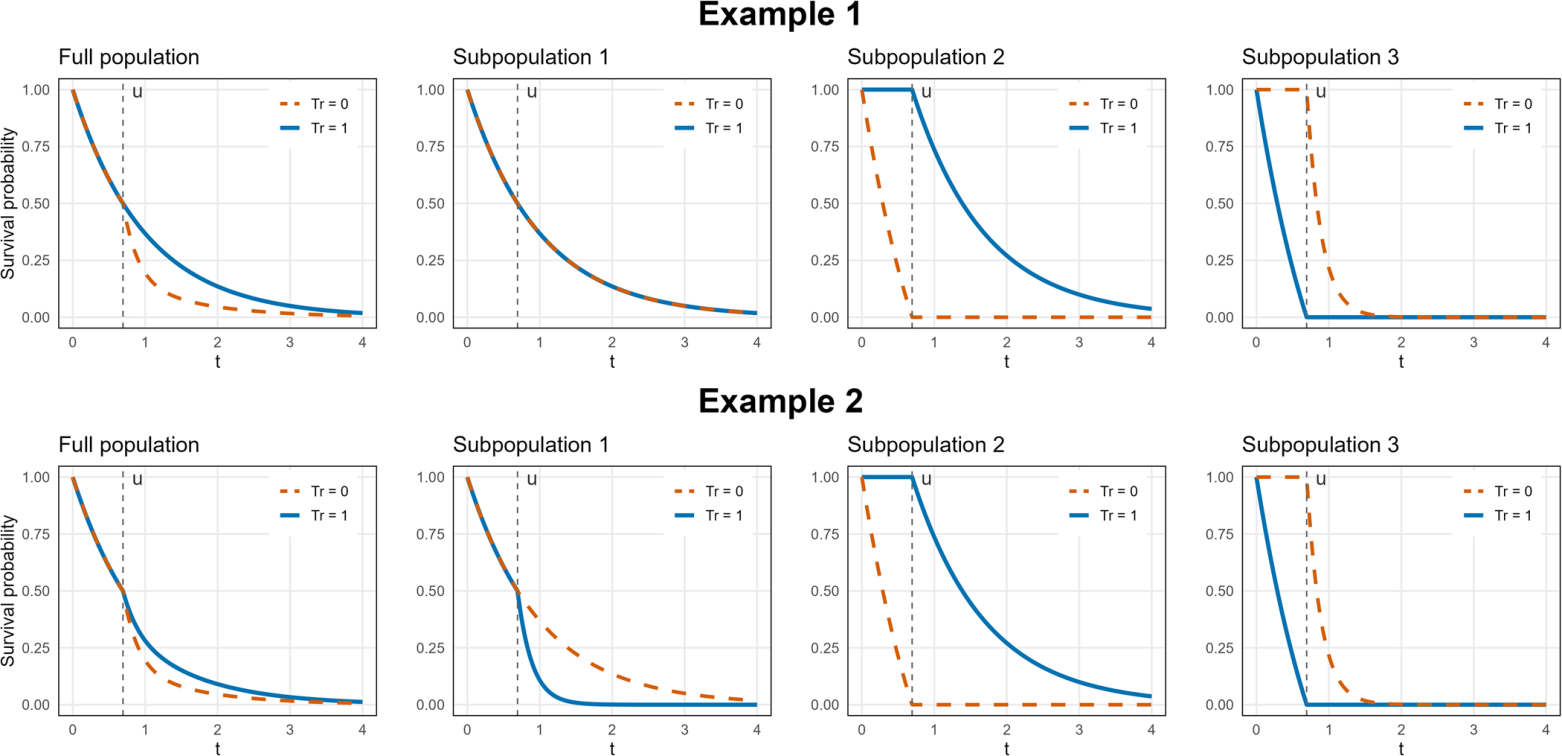
The first two examples described in Section 3.2. The left plot represents the survival functions with (solid blue line) and without treatment (dashed red line) for the full population. The three following plots illustrate the survival functions in the three subpopulations as described in the text.

In all examples, it is assumed that $T_{0}$ and $T_{1}$ are conditionally independent given by S, that is,

$$P\left(T_{0}\leq t_{0},T_{1}\leq t_{1}|S=s\right)=P\left(T_{0}\leq t_{0}|S=s\right)\;P\left(T_{1}\leq t_{1}|S=s\right),$$

for all s=1,2,3, $t_{0},t_{1}>0$. An illustration of Example 1 and Example 2 is given in Figure 1.

Example 1 For the first example, the distributions of $T_{0}$ and $T_{1}$ in the subpopulations s=1,2,3 are given by

$$\begin{array}{cc} S=1:\; & S_{0|S=1}\left(t\right)=S_{1|S=1}\left(t\right)=\exp\left(-t\right).\\ S=2:\; & S_{0|S=2}\left(t\right)=\left(2\;\exp\left(-t\right)-1\right)\;1_{\left\{t\leq u\right\}},\\ & S_{1|S=2}\left(t\right)=1_{\left\{t\leq u\right\}}+\left(2\exp\left(-t\right)\right)\;1_{\left\{t>u\right\}}.\\ S=3:\; & S_{0|S=3}\left(t\right)=1_{\left\{t\leq u\right\}}+\left(2^{5}\exp\left(-5t\right)\right)\;1_{\left\{t>u\right\}},\\ & S_{1|S=3}\left(t\right)=\left(2\;\exp\left(-t\right)-1\right)\;1_{\left\{t\leq u\right\}}. \end{array}$$

A straightforward calculation yields that the survival function of $T_{0}$ is

$$S_{0}\left(t\right)=\exp\left(-t\right)1_{\left\{t\leq u\right\}}+\frac{1}{3}\left(\exp\left(-t\right)+2^{5}\exp\left(-5t\right)\right)1_{\left\{t>u\right\}},$$

and the survival function of $T_{1}$ is

$$S_{1}\left(t\right)=\exp\left(-t\right).$$

It is not hard to see, that, for all t>u,

$$S_{0}\left(t\right)<S_{1}\left(t\right).$$

On the other hand, for all t≥u, it holds

$$P\left(T_{0}\geq t,T_{1}\geq t|S=2\right)\leq P\left(T_{0}\geq t|S=2\right)=0,$$

and

$$P\left(T_{0}\geq t,T_{1}\geq t|S=3\right)\leq P\left(T_{1}\geq t|S=3\right)=0.$$

Hence,

$$P\left(T_{0}\geq t,T_{1}\geq t\right)=P\left(T_{0}\geq t,T_{1}\geq t,S=1\right);$$

consequently the causal hazard ratio ${\text{HR}}_{C}\left(t\right)$ reduces to the causal hazard ratio in Subpopulation 1. Due to the conditional independence of $T_{0}$ and $T_{1}$ given S, this is just the regular hazard ratio in this subpopulation, and it follows that ${\text{HR}}_{C}\left(t\right)=1$ for all t≥u. Moreover, symmetry considerations yield that ${\text{HR}}_{C}\left(t\right)=1$ for t<u.

Example 2 For the second example, the distributions of $T_{0}$ and $T_{1}$ in the subpopulations s=1,2,3 are given by

$$\begin{array}{cc} S=1:\; & S_{0|S=1}\left(t\right)=\exp\left(-t\right),\\ & S_{1|S=1}\left(t\right)=\exp\left(-t\right)1_{\left\{t\leq u\right\}}+2^{4}\exp\left(-5t\right)1_{\left\{t>u\right\}}\\ S=2:\; & S_{0|S=2}\left(t\right)=\left(2\;\exp\left(-t\right)-1\right)\;1_{\left\{t\leq u\right\}},\\ & S_{1|S=2}\left(t\right)=1_{\left\{t\leq u\right\}}+\left(2\exp\left(-t\right)\right)\;1_{\left\{t>u\right\}}.\\ S=3:\; & S_{0|S=3}\left(t\right)=1_{\left\{t\leq u\right\}}+\left(2^{5}\exp\left(-5t\right)\right)\;1_{\left\{t>u\right\}},\\ & S_{1|S=3}\left(t\right)=\left(2\;\exp\left(-t\right)-1\right)\;1_{\left\{t\leq u\right\}}. \end{array}$$

The survival function of $T_{0}$ is again

$$S_{0}\left(t\right)=\exp\left(-t\right)1_{\left\{t\leq u\right\}}+\frac{1}{3}\left(\exp\left(-t\right)+2^{5}\exp\left(-5t\right)\right)1_{\left\{t>u\right\}},$$

and the survival function of $T_{1}$ is

$$S_{1}\left(t\right)=\exp\left(-t\right)1_{\left\{t\leq u\right\}}+\frac{1}{3}\left(2\exp\left(-t\right)+2^{4}\exp\left(-5t\right)\right)1_{\left\{t>u\right\}}.$$

By elementary considerations,

$$S_{0}\left(t\right)=S_{1}\left(t\right)\;\text{for}\;t\leq u,\;S_{0}\left(t\right)<S_{1}\left(t\right)\;\text{for}\;t>u,$$

demonstrating a survival benefit for treatment. On the other hand, using analogous arguments as for the first example,

$${\text{HR}}_{C}\left(t\right)=1\;\text{for}\;t<u,\;{\text{HR}}_{C}\left(t\right)>1\;\text{for}\;t\geq u.$$

Example 3 Finally, a third example can be constructed by removing Subpopulation 1 from either of the two examples above (that is, considering the distribution P(·|S≠1)). Then, for t≥u

$$P(T_{0}\geq t,T_{1}\geq t)=0,$$

that is neither ${\text{HR}}_{C}\left(t\right)$ nor any other causal effect measure at time t are meaningfully defined.

The reader may be skeptical about the validity of these examples, since values t>u are not part of the joint support of $T_{0}$ and $T_{1}$ in *each subgroup*. Yet specifying the support of $T_{0}$ and $T_{1}$ on a subgroup (or individual) level would inevitably imply assumptions about their dependence structure, which—as common in causal inference—is left unspecified in Assumptions 1.

### 3.3 Interpretation

The first finding is that the conventional hazard ratio does not necessarily represent a causal effect for a sensible subpopulation at risk at time u, even when the survival curves of the two survival times are equal up to time u. This implies, in particular, that the conventional hazard ratio HR and the causal hazard ratio ${\mathrm{HR}}_{C}$ do not necessarily coincide in this setting. Consequently, the interpretation of HR(u) as a treatment effect at time u is at least questionable.

While this is surprising and diametral to at least part of the literature, one may still argue that HR(u) has a straightforward causal effect interpretation in this setting—however on the population at baseline and not on any population at risk at time u. Taking Example 1 and assuming no knowledge about the subgroup affiliation, the survival probability with and without treatment is the same before u and better for treatment after u. Without any further information, a reasonable individual would prefer the treatment option for the reason that they expect a better survival after time u.

The second finding is that there are situations where the causal hazard ratio ${\mathrm{HR}}_{C}\left(t\right)$ does not represent a satisfying stand‐alone measure for the treatment effect at time t. The most striking may be Example 2, in which the survival with treatment is better for any point t>u, while the causal hazard ratio ${\text{HR}}_{C}\left(t\right)$ is greater than 1 indicating that treatment is harmful in the group $\left\{T_{0}\geq t,T_{1}\geq t\right\}$. While there is no fundamental problem with the interpretation of the causal hazard ratio per se (treatment is indeed harmful in this subgroup), summarizing the treatment effect over time with the causal hazard ratio alone is clearly not satisfying. Without any knowledge about the subgroup affiliation, any reasonable individual would again prefer treatment over no treatment, while interpreting the causal hazard ratio as the (and not one particular) treatment effect at time t would suggest otherwise. In fact, it appears that in this situation the causal interpretation of the conventional hazard ratio (for the population at baseline) is more straightforward than the interpretation of the causal hazard ratio.

Consequently, the use of the causal hazard ratio ${\mathrm{HR}}_{C}\left(t\right)$ as an estimand reflecting the treatment effect at time t is questionable; even more so since—by Example 3—it may not even exist in a meaningful way. It is evident that these arguments are not restricted to the causal hazard ratio and extend to any causal effect on subsets of $\left\{T_{0}\geq t,T_{1}\geq t\right\}$. Altogether the findings in Sections 2 and 3 suggest that, without making further assumptions on the dependence structure of $T_{0}$ and $T_{1}$, no satisfying causal estimand for a population treatment effect at time t exists!

## 4 Toward Time‐Dependent Treatment Effects

There are essentially two possible responses to the shortcomings discussed above. The first is to conclude that a useful and coherent time‐dependent treatment effect cannot be defined and advocate against any attempt to estimate or interpret it. The second approach, that will be discussed for the remainder of this paper, is to make additional structural assumptions that deliver a well‐defined estimand at time t and enable some “degree of inference”. Such assumptions unavoidably constrain the joint distribution of $T_{0}$ and $T_{1}$ and hence remain untestable—yet context and domain knowledge may render them plausible; for instance, as Martinussen et al. (2020) argued, $T_{0}$ and $T_{1}$ are usually expected to be positively correlated.

A future line of methodological research may hence work on deriving various assumptions and corresponding fully coherent causal estimands enabling inference for time‐dependent treatment effects. The choice of estimand should reflect the scientific question at hand and need not coincide with the causal hazard ratio ${\mathrm{HR}}_{C}\left(t\right)$. Under certain assumptions it might even be possible to define an estimand for all individuals still at risk at time t—for example, the hazard ratio that would emerge if everyone were re‐randomized at that moment. Stronger, more explicit assumptions could allow point identification and consistent estimation, whereas weaker conditions may yield only partial identification such as upper and lower bounds.

Illustrative results already exist. Martinussen (2022) showed that if HR(t)≡HR<1 is constant and the joint distribution of $T_{0}$ and $T_{1}$ is induced by a frailty, then

$${\mathrm{HR}}_{C}\left(t\right)<\mathrm{HR}\left(t\right)<1.$$

Conversely, Section 3.4 of the corresponding arXiv preprint (Martinussen et al. 2018) specifies conditions that create a one‐to‐one relationship between ${\mathrm{HR}}_{C}\left(t\right)$ and HR(t), thereby allowing consistent estimation of ${\mathrm{HR}}_{C}\left(t\right)$.

The use of inference methods for time‐dependent treatment effects in practice should always be guided by considerable caution. They must be accompanied by sensitivity studies spanning multiple assumption sets, and any results should be regarded as exploratory until they are confirmed, for instance by a properly designed trial with change of treatment at time t.

## 5 Concluding Remarks

As pointed out by Beyersmann et al. (2025), time‐to‐event data is special. While censoring—discussed at length in that paper—is one such peculiarity, it is by no means the only one. Because of their temporal nature, the analysis of time‐to‐event endpoints differs fundamentally from that of many other continuous endpoints—such as clinical measurements taken at fixed time points. For example, *conditional on survival up to time* t, it is of substantial interest to know if the event happens in the near future (say in the interval (t,t+Δt]), a question that has no meaningful analogue for outcomes like blood‐pressure readings. In modeling event or transition times, it has proven useful—both for theoretical development and practical interpretation—to frame the process in terms of infinitesimal event probabilities (hazards or intensities), even in settings where censoring is of little or no concern, such as in queuing theory (Kleinrock 1975, p. 48) or Hawkes processes (Hawkes 1971). Aalen (1989, p. 908) provided an insightful discussion of this issue connecting the concept of intensities to the so‐called “French School of probability”; in particular, he states that “by giving the intensity concept a primary place, one recognizes that survival [...] data” are “not just any measurements which happen to be censored occasionally, but are measurements of times and should be analyzed as such.”

Because hazard contrasts compare the instantaneous event rate given survival to time t, they are natural candidates for the treatment effect at time t—an inherently conditional quantity that can hence only be assessed using conditional tools. Although we agree with Beyersmann et al. (2025) that unconditional measures such as survival probabilities and their contrasts are extremely useful—and ought to be reported “much more routinely”, they are not suited for this type of question. Unfortunately, it is precisely the conditional nature that impedes the definition and identification of time‐dependent treatment effects. Conditioning on survival up to time t breaks the original comparability of randomized groups, making it impossible to define or identify a time‐dependent treatment effect without further assumptions.

From a mathematical perspective, imposing usual assumptions for continuous or time‐to‐event endpoints in causal inference, these problems appear very discouraging; a meaningful and coherent treatment effect at time t may not even exist and identification of candidates such as the causal hazard ratio is virtually impossible. A practitioner might judge this verdict to be too pessimistic—and may argue that an experienced bistatistician being aware about the pitfalls described in the literature would be able to give at least an educated guess for a treatment effect, even if only in a naive or intuitive sense.

By introducing additional, medically plausible assumptions to constrain dependence structures, future inference procedures for time‐dependent effects may be built on a rigorous mathematical foundation. As discussed before, obtained results may still be rather crude and should be interpreted with caution. However, it may happen that one must make a decision about a time‐dependent treatment effect— for example, when determining the timepoint for a change of the treatment regime in the planning phase of a clinical trial. In these situations, we firmly believe that an educated guess should always be chosen over a wild one.

## Conflicts of Interest

The author declares no conflicts of interest.

## Data Availability

Data sharing not applicable to this paper as no datasets were generated or analyzed during the current study.

## Appendix A

In Section 2.2, it is mentioned that the given definition for a causal effect is somewhat restrictive since both the functional and the contrast function are assumed to be real‐valued. In the following, an attempt for a more general definition is made that avoids the use of two different functionals and defines the causal effect directly as a contrast on the induced probability measures of the two counterfactuals. For the following definition, let G be a group with neutral element e. Further denote the inverse element of g∈G as $g^{-}$.
Definition A.1 Let P denote a set of probability measures on a measurable space (Ω,F). Let $Y_{0}$ and $Y_{1}$ denote two measurable functions denoting counterfactual outcomes on (Ω,F) with $P_{Y_{0}},P_{Y_{1}}\in P$. Let ψ:P×P→G be a functional satisfying

i. For all Q∈P, ψ(Q,Q)=e.
ii. For all $Q_{1},Q_{2}\in P$, $\psi \left(Q_{1},Q_{2}\right)=\psi {\left(Q_{2},Q_{1}\right)}^{-}$.

Then, $\psi (P_{Y_{1}},P_{Y_{0}})$ is called a **population causal effect** for comparing $Y_{1}$ and $Y_{0}$ under the probability distribution P.

There may be settings, in which the direction of the effect is of little interest—it may then make sense to allow for a definition, say of an *unsigned causal effect*, in which G is replaced by a semigroup with neutral element and the antisymmetry condition in (ii) by a symmetry condition.
